# Diagnosis of bilateral diffuse uveal melanocytic proliferation unveils primary gastric adenocarcinoma: a case report

**DOI:** 10.1186/s12886-020-01376-2

**Published:** 2020-03-19

**Authors:** Mingyue Luo, Zhe Chen, Yaping Luo, Lin Zhao, Rongping Dai, Yong Zhong

**Affiliations:** 1 Department of Ophthalmology, Peking Union Medical College Hospital, Chinese Academy of Medical Sciences, Beijing, 100730 China; 2 Key Lab of Ocular Fundus Disease, Chinese Academy of Medical Sciences, Beijing, 100730 China; 3 Department of Nuclear Medicine, Peking Union Medical College Hospital, Chinese Academy of Medical Sciences, Beijing, 100730 China; 4 Department of Oncology, Peking Union Medical College Hospital, Chinese Academy of Medical Sciences, Beijing, 100730 China

**Keywords:** BDUMP, Gastric adenocarcinoma, Paraneoplastic syndrome

## Abstract

**Background:**

Bilateral diffuse uveal melanocytic proliferation (BDUMP) is an extremely rare paraneoplastic syndrome, with most cases reported as secondary to female urogenital and male lung malignancies. We reported this case of BDUMP patient whose primary malignancy was gastric adenocarcinoma verified with gastroscopy and subsequent pathological test.

**Case presentation:**

A patient complaining blurred vision was suspected of bilateral diffuse uveal melanocytic proliferation (BDUMP), due to bilateral round oval patches at the posterior pole and cardinal signs in retinal angiography. Malignancy screening was suggested, and pathological report from gastroscopy confirmed the primary lesion as gastric adenocarcinoma. The patient chose palliative care due to late stage and unresectable nature of the malignancy.

**Conclusions:**

Identifying BDUMP warrants further investigation of a primary malignancy. Our case provided evidence for the link between gastric adenocarcinoma and BDUMP.

## Background

Bilateral diffuse uveal melanocytic proliferation (BDUMP) is a rare paraneoplastic syndrome (PNS) affecting the eye, with around 60 cases reported [1]. There are five cardinal signs: (1) multiple, round or oval, subtle, patches at the level of the retinal pigmented epithelium (RPE) in the posterior fundus; (2) multifocal areas of early hyper-fluorescence corresponding with these patches; (3) multiple, slightly elevated, pigmented and non-pigmented uveal melanocytic tumors, as well as evidence of diffuse thickening of the uveal tract; (4) exudative retinal detachment; and (5) rapid progression of cataract [2]. It is thought either a substance secreted by the tumor or an antibody stimulated by the tumor, that causes benign proliferation of choroidal melanocytes [1]. Female urogenital (69%) and male lung carcinomas (52%) were reported more often, with sporadic cases including pancreatic, esophageal, breast, hepatocellular, Bartholin gland and renal cell carcinoma and central nervous system lymphoma [1]. Herein, we reported this case of BDUMP patient whose primary malignancy was gastric adenocarcinoma verified with gastroscopy and subsequent pathological test. This report was organized in adherence to CARE guidelines.

## Case presentation

A 50-year-old Chinese male presented with bilateral blurred vision for 3 months. Nine months earlier, he experienced pulmonary embolism and lower limb venous thrombosis, and was diagnosed with antiphospholipid antibody syndrome (APS). He had lost 10 kilograms in the past 9 months. No other gastric or constitutive symptoms were reported.

**Fig. 1:**
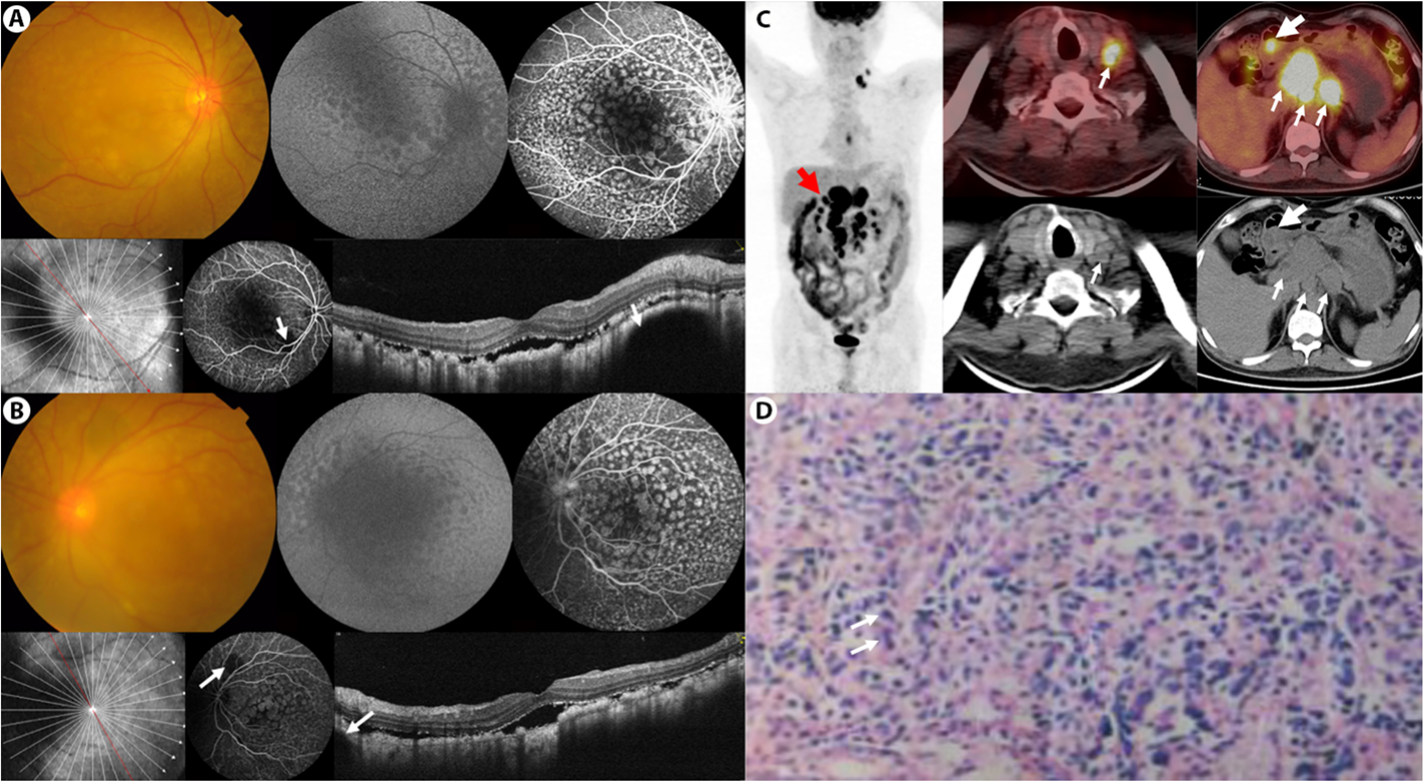
Multimodal imaging of bilateral diffuse uveal melanocytic proliferation secondary to gastric adenocarcinoma. **a** and **b** Fundus photography (top left) showed bilateral diffuse oval yellow patches in the posterior pole, corresponding to hypo-fluorescence in autofluorescence (top middle) and hyper-fluorescence in the early and late phases of fundus fluorescein angiography (top right) and indocyanine green angiography (ICGA, bottom middle). Notice the choroidal lesions (white arrows) indicating choroidal melanocytic proliferation in ICGA and spectral domain optical coherence tomography (bottom right). **c**^18^F-FDG PET/CT showed an FDG-avid lesion in the gastric antrum (big arrow), and multiple hypermetabolic lymph nodes in perigastric, retroperitoneal, mediastinal and left supraclavicular region (small arrows). **d** Haematoxylin-eosin staining of the gastric lesion, confirming gastric adenocarcinoma. The neoplastic cells with most deeply-stained nuclei were diffusely distributed (white arrows), mixed with lymphocytes and epithelium

On examination, his best corrected visual acuity (BCVA) was 20/200 OU. Anterior chamber was basically normal except for moderate cataract in both eyes. Fundus examination showed bilateral diffuse oval yellow patches in the posterior pole (Fig. 1a and b, top left), corresponding to a classical giraffe sign, namely hypo-fluorescence in autofluorescence (AF, Fig. 1a and b, top middle) and hyper-fluorescence in the early and late phases of fundus fluorescein angiography (FFA, Fig. 1a and b, top right) and indocyanine green angiography (ICGA) (Fig. 1a and b, bottom middle), with late phase pinpoint leakage. Spectral domain optical coherence tomography (SD-OCT, Fig. 1a and b, bottom right) B-scan well-depicted a mosaic pattern of RPE alterations between irregular thickening and atrophy. Blocked fluorescence on ICGA due to choroidal lesions was also noticed (Fig. 1a and b, white arrows). Based on these typical findings, the patient was diagnosed with BDUMP, and malignancy screening was strongly recommended.

Blood tumor markers reported as: CA19–9795.0 U/ml, CA125 3770.0 U/ml, Cyfra 211 57.6 ng/ml, CA242 > 150.000 U/ml, NSE 46.1 ng/ml. ^18^F- fluorodeoxyglucose (FDG) positron emission tomography–computed tomography (PET/CT) (Fig. 1c) showed an FDG-avid lesion in the gastric antrum (Fig. 1c, big arrow), and multiple hypermetabolic lymph nodes (Fig. 1c, small arrows) were also noted in perigastric, retroperitoneal, mediastinal and left supraclavicular region, suggestive of gastric malignancy with distant lymph node metastasis. Based on these findings, gastroscopy was ordered. Pathological diagnosis (Fig. 1d) reported as poorly differentiated adenocarcinoma. The patient was finally diagnosed with BDUMP and secondary APS due to gastric adenocarcinoma. Systemic chemotherapy was suggested, but after evaluation, the patient’s systemic condition was too poor to tolerate any chemotherapy. After consideration, the patient chose palliative care out of the late stage and unresectable nature of the malignancy and economic reasons.

## Discussion and conclusions

BDUMP is an extremely rare paraneoplastic syndrome affecting the eye secondary to a primary malignancy, which can be ocular as well as systemic. We reported a case of BDUMP secondary to gastric adenocarcinoma, verified with pathological staining. Gastric adenocarcinoma was rarely reported to be associated with BDUMP. Dolz-Marco et al. [3] reported one delayed onset BDUMP case 17 years after total gastrectomy for gastric adenocarcinoma, with no evidence of primary cancer recurrence or second malignancy. Our case validated the association of gastric adenocarcinoma and BDUMP.

Despite various origins of primary malignancies, the mechanism of BDUMP is considered to be associated with a serum factor in patients’ IgG fraction, namely cultured melanocyte elongation and proliferation (CMEP) factor [4]. Hepatocyte growth factor (HGF) and anti-retinal autoantibodies to α-HGF were also suggested as an alternative etiology [5].

Treatment of BDUMP primarily targets the primary malignancies, including local resection, radiation and systemic chemotherapy. Since systemic factors elicited by primary malignancies is considered involved in the pathogenesis of BDUMP, this could possibly explain the improvement of visual symptoms in some cases after these treatments targeting the malignancy [1]. Plasmapheresis can theoretically remove plasma CMEP, but with variable effectiveness [6]. Intravitreal anti- vascular endothelium growth factor (VEGF) agents were proven effective in some cases with intra-retinal fluid [3]. Other interventions such as ocular radiation, sub-retinal fluid drainage, corticosteroids were generally unsuccessful [6]. The prognosis of BDUMP is extremely poor, with 15.6 months’ median survival and in some exceptional cases, 4 to 9 years [7], due to the dissemination of the primary malignancy.

In summary, identifying BDUMP warrants further investigation of a primary malignancy. Our case provided evidence for the link between gastric adenocarcinoma and BDUMP.

## Data Availability

Not applicable.

## Abbreviations

BDUMP: Bilateral diffuse uveal melanocytic proliferation
PNS: Paraneoplastic syndrome
APS: Antiphospholipid antibody syndrome
RPE: Retinal pigmented epithelium
BCVA: Best corrected visual acuity
AF: Autofluorescence
FFA: Fundus fluorescein angiography
ICGA: Indocyanine green angiography
SD-OCT: Spectral domain optical coherence tomography
FDG: Fluorodeoxyglucose
PET/CT: Positron emission tomography–computed tomography
CMEP: Cultured melanocyte elongation and proliferation
HGF: Hepatocyte growth factor
VEGF: Vascular endothelium growth factor

## References

[1] Klemp K, Kiilgaard JF, Heegaard S, Norgaard T, Andersen MK, Prause JU (2017). Bilateral diffuse uveal melanocytic proliferation: case report and literature review. Acta Ophthalmol.

[2] Gass JD, Gieser RG, Wilkinson CP, Beahm DE, Pautler SE (1990). Bilateral diffuse uveal melanocytic proliferation in patients with occult carcinoma. Arch Ophthalmol (Chicago, Ill: 1960).

[3] Dolz-Marco R, Vilaplana F, Gallego-Pinazo R, Freund KB (2017). Delayed-onset bilateral diffuse uveal melanocytic proliferation associated with gastric adenocarcinoma. Retin Cases Brief Rep.

[4] Miles SL, Niles RM, Pittock S (2012). A factor found in the IgG fraction of serum of patients with paraneoplastic bilateral diffuse uveal melanocytic proliferation causes proliferation of cultured human melanocytes. Retina (Philadelphia, Pa).

[5] Niffenegger JH, Soltero A, Niffenegger JS, Yang S, Adamus G. Prevalence of hepatocyte growth factor and autoantibodies to alpha-HGF as a new etiology for bilateral diffuse uveal melanocytic proliferation masquerading as neovascular age-related macular degeneration. J Clin Exp Ophthalmol. 2018;9(4). 10.4172/2155-9570.1000740. 10.4172/2155-9570.1000740 PMC6201244 30370177

[6] Jaben EA, Pulido JS, Pittock S, Markovic S, Winters JL (2011). The potential role of plasma exchange as a treatment for bilateral diffuse uveal melanocytic proliferation: a report of two cases. J Clin Apher.

[7] Mittal R, Cherepanoff S, Thornton S, Kalirai H, Damato B, Coupland SE (2015). Bilateral diffuse uveal melanocytic proliferation: molecular genetic analysis of a case and review of the literature. Ocul Oncol Pathol.

